# How to Promote Gerotranscendence in Older Adults? A Scoping Review of Interventions

**DOI:** 10.1177/07334648231169082

**Published:** 2023-04-13

**Authors:** Taiane Abreu, Lia Araújo, Oscar Ribeiro

**Affiliations:** 1 Department of Education and Psychology, 56062 University of Aveiro (DEP.UA), Portugal; 2 Center for Health Technology and Services Research (CINTESIS), Aveiro, Portugal; 3 School of Education, 112016 Polytechnic Institute of Viseu (ESEV.IPV), Portugal

**Keywords:** gerotranscendence, mental health, older adults, interventions, well-being

## Abstract

Gerotranscendence is a psychosocial theory that proposes older adults experience a mindset shift in multiple dimensions (cosmic, coherence, solitude), associated with different constructs, such as life satisfaction and well-being. Increasing studies are employing gerotranscendence, but the practical aspects of how gerotranscendence can be developed are still underexplored. This review involved an assessment of the size and scope of the existing literature on interventions related to gerotranscendence and their effect on participants’ well-being. Six databases were searched, and eight studies were selected: five were observational, while three were randomized control trials. Findings showed that gerotranscendence can be developed through weekly thematic encounters. Moreover, the interventions examined in the selected studies were described as having positively impacted participants’ mental health and life satisfaction. Future studies should explore gerotranscendence interventions using a rigorous methodology and long-term follow-up data to add consistency to these findings.

**What this paper adds**

• This manuscript presents the first scoping review to explicitly explore gerotranscendence interventions.
• The types and organization of interventions are synthesized, providing a critical viewpoint on practical aspects of gerotranscendence development.
• This review highlights the importance of promoting older adults’ well-being and presents an innovative and promising type of intervention.

**Applications of study findings**

• The results identify the main themes considered in gerotranscendence interventions and how they are addressed in practical terms.
• Providing an open space where participants can discuss core aspects of the gerotranscendence theory and related topics, such as mental health issues and life satisfaction in late life, seems most effective for increasing gerotranscendence, mental health, and life satisfaction levels.
• By mapping information on multiple types of interventions, this review highlights ways for future studies to address older adults’ mental health issues through gerotranscendence with more consistency.

## Introduction

Gerotranscendence is an adaptative theory of aging that postulates that a mindset shift occurs in late life in three dimensions: cosmic, where individuals feel more connected to the universe, nature, and past generations; coherence, related to the development of ego integrity and a decrease in self-centeredness; and solitude, involving changes in the meaning of relationships, when individuals become more selective with company they keep and need more time for contemplation (Jewell, 2014; Rajani & Jawaid, 2015b).

Qualitative studies based on gerotranscendence theory have been published to enhance our understanding of gerotranscendence and its characteristics (George & Dixon, 2018; Tornstam, 2005). Additionally, a gerotranscendence scale (GST) was developed to measure older adults’ level of gerotranscendence that made quantitative studies possible (Tornstam, 2011). With the use of an objective scale, Tornstam (2005), who originally proposed the gerotranscendence theory, verified cultural differences, relatable constructs (e.g., life satisfaction), and possible modifiers of gerotranscendence in older adults, such as life circumstances, past crises, and gender roles.

Tornstam (2011) suggested that only about 20% of the population would spontaneously reach high levels of gerotranscendence, while the mindset shift for many individuals would transpire more slowly or even be blocked, for various reasons. A slower process may be explained by the individual’s life circumstances and other identified modifiers; the block, on the other hand, occurs because individuals in late life continue to hold values, beliefs, and interests, as they do in midlife, that can impair their maturation process and hinder their appreciation for the transformations that accompany growing older (Tornstam, 2005, 2011). This resistance to change can facilitate the development of depression and anxiety, as it may be related to the idea of despair, the fear of death, and/or a maladaptive interpretation of life events, especially difficult ones (Jeffers et al., 2020; Tornstam, 2011).

Hence, individuals must shift their mindsets to achieve gerotranscendence to embrace the changes of growing older and to more maturely interpret life. This mindset and behavior are understood as developmental, so the process of acquiring gerotranscendence characteristics can differ for each individual (Tornstam, 2005). The idea of multiple pathways to achieving gerotranscendence represents a versatile side of the theory, indicating people from diverse cultures and life contexts can experience the phenomenon as long as its dimensions (cosmic, coherence, and solitude) are contemplated. Moreover, gerotranscendence has been associated with life crises, negative life events, and religious beliefs (Abreu et al., 2021, 2022; Read et al., 2014; Tornstam, 2005), meaning life experiences that promote existential reflections, as these types of events do, are more prone to stimulate gerotranscendence development (van Rhyn et al., 2022).

The relation between existential reflections and gerotranscendence (van Rhyn et al., 2022), the multiple pathways to achieving it (Tornstam, 2005), and older adults’ natural identification with the theory (Heinz et al., 2017) suggest interventions can help develop and/or enhance gerotranscendence (Sánchez Cabaco and Fernández Mateos, 2019). Such interventions may also improve older adults’ mental health, as higher levels of gerotranscendence has been associated with a greater degree of well-being, purpose in life, and life satisfaction and with lower levels of depression, anxiety, and fear of death (Braam et al., 2006, 2016; George & Dixon, 2018; Raeesi Dehkordi et al., 2020; Wang, 2011; Wang et al., 2015).

Tornstam (2005) identified some exercises that may promote higher levels of gerotranscendence, while additional authors (e.g., Rajani and Jawaid, 2015a) proposed other relevant interventions. However, the literature lacks a structured review of the existing evidence on gerotranscendence interventions and, thus, remains unclear on the characteristics of effective interventions, such as the way they are executed, the way gerotranscendence is addressed, and the associated outcomes. Systematizing these details can provide a greater understanding of whether and, if so, how gerotranscendence links to the benefits outlined in previous exploratory studies in practical terms and would add to existing evidence on interventions to support older adults’ mental health, well-being, and life satisfaction (Sánchez Cabaco and Fernández Mateos, 2019; Raeesi Dehkordi et al., 2020).

As the lack of mapping of available information on gerotranscendence can impede knowledge advancements in the field, the present review assessed the potential size and scope of available research on interventions related to gerotranscendence and their effect on participants’ well-being.

## Methods

The Preferred Reporting Items for Systematic Reviews and Meta-Analyses (PRISMA) was employed for this scoping review (Page et al., 2021) (See Appendix 1 for more details of PRISMA checklist).

### Inclusion and Exclusion Criteria

The studies included presented gerotranscendence interventions (programs or activities) that focused on adults aged 65 years and older or compared results for middle-aged and older adults with mild to no cognitive impairment. The designs of the studies included were randomized control trials (RCTs) or non-RCTs that performed any type of intervention related to gerotranscendence and evaluated gerotranscendence as an outcome. Quantitative and qualitative studies published in peer-reviewed journals or featured in academic theses/dissertations written in English, Spanish, or Portuguese were included. Studies that involved interventions targeting formal and informal caregivers, that did not present a clear objective to promote gerotranscendence, and that did not report on an intervention, program, or activity based on gerotranscendence were excluded.

### Search Strategy and Screening

Searches of the PubMed, SCOPUS, PsycInfo, Cochrane, Web of Science, and for theses and dissertations, ProQuest databases were conducted January 7–20, 2022, with results updated November 28, 2022, using the following search terms, which had to appear in the title, abstract, or keywords: gerotranscendence* AND program OR practice OR intervention OR activity OR guideline OR session OR randomized control trial OR technique. To increase the probability of finding sufficient articles, a forward search was also performed, during which the reference lists from all eligible studies were examined to expand the review corpus. No restrictions were placed on the date of publication.

### Data Extraction

Data extraction was based on the Template for Intervention Description and Replication (TIDieR; Hoffmann et al., 2014), through which the following details from each study was compiled for analysis: country where study was performed; study design, goal, and rationale; intervention provider/implementer; intervention process; intervention materials and procedure/session content; frequency and duration of each session; any modification or personalization of the intervention; participant characteristics; participant attendance; and major results presented by authors. Then, a thematic analysis was performed to map the main themes addressed in each article’s discussion on the study outcomes (Ritchie & Spencer, 1994). For this, the first author conducted a preliminary analysis of the studies included and executed an initial thematic framework according to the main outcomes reported and defined specific themes based on the analysis. Next, the first and second authors independently assigned each study to a primary theme and then compared their results: discrepancies were discussed and resolved with the third author.

### Risk of Bias Assessment and Critical Appraisal

The Risk of Bias in Non-Randomized Studies—Interventions (ROBINS-I) instrument (Sterne et al., 2016) was used to identify the risk of bias in each study as low, moderate, serious, critical, or unable to determine with information available. The first and second authors each completed the instrument separately and then compared results and discussed any discrepancies in their rankings to reach a consensus; when a consensus could not be reached, the third author determined the risk of bias for that study.

Next, the interventions were critically appraised based on the Grading of Recommendations Assessment, Development, and Evaluation (short GRADE) tool (Brignardello-Petersen et al., 2020; Goldet & Howick, 2013), which includes five domains: (1) overall risk of bias (randomization, allocation concealment, blinding, incomplete outcome data, selective reporting), (2) inconsistency (significant and unexplained variability in results), (3) indirectness (indirect comparison of population, outcome, or intervention), (4) imprecision (wide confidence intervals that jeopardize data quality), and (5) publication bias (lack of studies with “negative” findings or commercially funded sources). Each category was rated as unclear, not serious, serious, or very serious; the quality of evidence was rated as very low, low, moderate, or high.

## Results

### Study Characteristics

**Figure 1.**
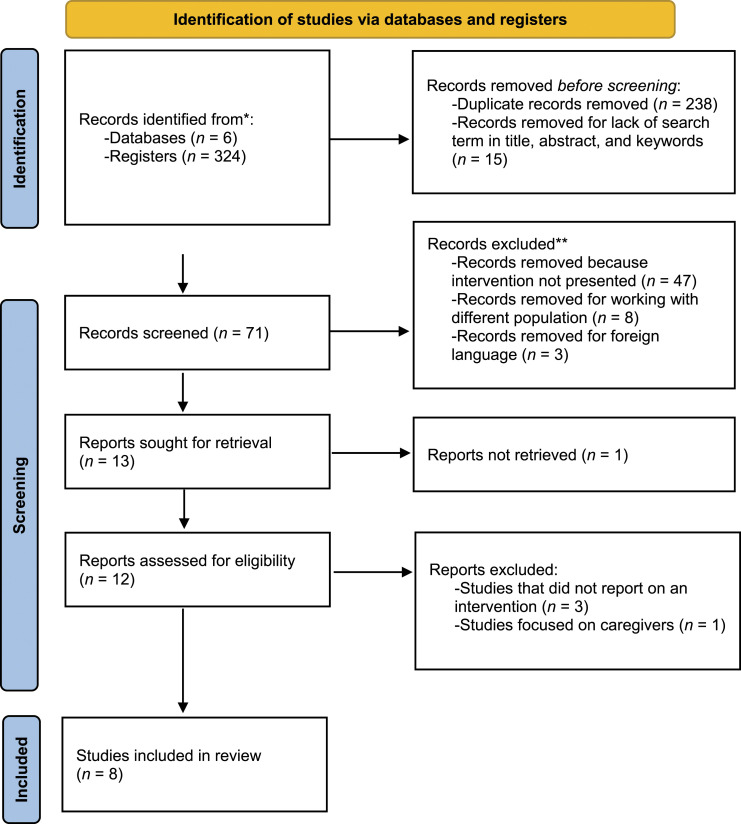
PRISMA selection of studies for the review.

Initially, 324 publications were found. After eliminating duplicates and articles with no search terms appearing in the title, abstract, or keywords, 71 papers were selected. Next, a trial was performed that removed 63 of the 71 studies from consideration according to the inclusion and exclusion criteria (Figure 1). Hence, eight studies were included in this review (Table 1). The publication dates ranged from 2005 to 2019, with four studies published in the last decade. Study sites included Sweden (*n* = 3), Taiwan (*n* = 2), United States (*n* = 2), and China (*n* = 1). Participants ranged in age from 54 to 99 years old. Six studies involved interventions with community-dwelling participants, and two with institutionalized older adults. Three studies used control groups, while the other five did not present any comparator. To evaluate gerotranscendence levels, the studies relied on a gerotranscendence scale (*n* = 3), semi-structured interviews (*n* = 3), participants’ artwork (*n* = 1), a spiritual life map along with an ego-integrity scale (*n* = 1).

**Table 1.** Summary of Findings Based on the Template for Intervention Description and Replication (TIDieR).

Study	Country	Study Design	Intervention Goal	Materials	Session Content Summary	How Intervention was Organized	Participant Characteristics	Analysis Used	Summary of Findings	Themes Identified
Chen et al., 2019	Taiwan	Randomized Control Trial (RCT)	To implement and evaluate feasibility and effect of a multidimensional support program (MSP) on gerotranscendence and depression in community-dwelling older adults. Control group: no intervention	Gerotranscendence scale (GST Scale), Geriatric Depression Scale—Short Form (GDS-SF) feasibility measures, visual analogue scale (VAS)	Drawing life discussion and sharing of experiences; understanding of cosmic domain; understanding of coherence domain and relationships with others in later life; how to overcome depression in later life; concept of ego integrity and despair	8 sessions of 60 minutes comprising three stages: 30 min of knowledge education; 15 min of interaction activity; and 15 min of experience sharing and discussion	91 participants completed the study; the mean age was 73.8 years old(*SD* = 5.9), ranging from 65 to 88 years of age	Mixed-model repeated measures ANOVA and mediation analysis to examine the changes in outcome measures over time and the underlying mechanisms	Participants of the MSP presented higher levels of gerotranscendence, especially in the cosmic domain, when compared with control group. For depression, although there were significant differences between groups, there was no interaction effect after the intervention	Mental health
Duan et al., 2016	China	RCT	To explore Tai Chi’s association with TA and GT and correlations between TA and GT in older adults from community. Control group: no intervention	GST Scale and Telomerase Activity (TA) measures (blood samples)	24 Tai Chi postures	Sessions of 60 minutes, 5 times a week for 6 months	43 participants in the intervention group; the mean age was 59.58 (*SD* = 5.6)	Independent-samples t-tests and ANOVA to compare the pre- and post-test scores of the intervention and control groups	Tai Chi improves the GT in participants. However, the improvement in TA and GT may instead require long-term Tai Chi exercises. Tai Chi training could promote a sense of purpose and direction in life	Physical health and purpose in life
Molinari et al., 2013	US	Observational study	To explore the meaning of spirituality in the lives of older adults across the lifespan through a spiritual life review and to understand its influence on levels of ego integrity and gerotranscendence	Spiritual Life map, interviews about Spiritual Life Review	Drawing a spiritual life map, conducting in-depth interviews based on the spiritual life review, and completing the post-test ego integrity scale	Researchers met with participants approximately 5 times: 1—meet and greet, 2—initial meeting, 3—SPMSQ, 4 spiritual life map, and 5—an interview based on spiritual life review	18 participants concluded the study; the mean age was 83.55, ranging from 69 to 92 years of age	Qualitative content analysis to identify themes and patterns in the participants’ narratives	Completing a spiritual life review provided an opportunity to confirm gerotranscendence as participants shared significant life events from childhood up to their current age that have influenced their perspectives on life	Personal growth, spirituality, and purpose in life
Stephenson, 2013	USA	Observational study	To (a) foster artistic identity, (b) activate a sense of purpose and motivation, (c) use art as a bridge to connect with others, and (d) support movement toward attainment of gerotranscendence	Art supplies	Thematic suggestions challenged participants with a question or opportunity to explore an aspect of their lives, leaving the choice of art materials and form of expression to the artist	Weekly sessions for 2 hours for over 6 years	Participants’ age ranged from 58 to 99 years of age	Narrative synthesis approach to identify common themes and trends across the studies	Observations during the CATS program suggest that making art contributes to satisfaction, self-esteem, and wisdom. These strengths characterize gerotranscendent behavior	Wisdom, satisfaction with life, personal growth, and purpose in life
Wadensten, 2005	Sweden	Observational study	To introduce the theory of gerotranscendence to a group of older adults	Structured sessions	Brief introduction to the theory of gerotranscendence; women’s opinions of growing older; discussion of a video about gerotranscendence	Six sessions of 1.5 hours for 6 weeks	Six women, ranging from 68 to 80 years of age	Qualitative content analysis to identify themes and patterns in the participants’ narratives	All women experienced aging that was in some way in line with theory’s description. Some signs—namely, “connection to earlier generations,” “life and death,” “subject of rejoicing,” “self-confrontation,” “rediscovery of the child within,” “ego-integrity,” “changed meaning and importance of relations,” and “everyday wisdom”—were described by all women	Wisdom and personal growth
Wadensten & Hagglund, 2006	Sweden	Observational study	To investigate older adults’ experiences with participating in a reminiscence group with a gerotranscendental perspective	Interviews and structured sessions	Introduction of the idea of reminiscence; memories of early childhood; enjoyments during younger life; discussion about technical innovations, and about most important people in life	8 sessions with 1.5 hour long	Eight people (two men and six women), ranging from 74 to 85 years of age	Qualitative content analysis to identify themes and patterns in the participants’ narratives	All participants believed they had reminisced and thought much more about their childhood. Three quite different views emerged of the recall experience and effects of participating in the reminiscence group: “An activity like any other; an activity that led to thoughts about memories from life or an activity that influenced my thoughts about life.”	Wisdom and personal growth
Wadensten, 2009	Sweden	Observational study	To investigate older adults’ experiences with participating in a dream-coaching group based on Jungian psychology	Interviews and structured sessions	Presentation about the theoretical foundation of dream coaching, followed by moments of sharing dreams and asking questions between participants	8 sessions with duration of 2 to 3 hours. And interviews with the participants were individually conducted by the researcher 6–10 weeks after the dream groups had been concluded	A total of 22 persons (3 men and 19 women), ranging in age from 64 to 86 years	Qualitative content analysis to identify themes and patterns in the participants’ narratives	The arrangement of dream-coaching groups for older people could be a way to promote personal development using this type of intervention	Personal growth
Wang et al., 2011	Taiwan	RCT	GT support group intervention could increase GT perspective, prevent the progression of depression, and enhance life satisfaction in elderly residents in the long term. Control group: no intervention	GST Scale, GDS-SF, and LSS	Sharing perspective and experience of universe transcendence, self-transcendence, and social transcendence; sharing worries of self, self-management of life events, how to adapt GT, and alleviate a depressed mood; sharing ways to improve life satisfaction; sharing perspectives of end of life, and fear of death	4 presential experimental groups, for 60 minutes, for 8 weeks. Each session had four phases: discussion (20 min) GT presentation (15 min); discussion supported by GT (15 min); and feedback (10 min)	35 participants in the experimental group; the mean age was 80.5, ranging from 65 to 95 years of age	Repeated measures ANOVA and mediation analysis to compare the pre- and post-test scores of the intervention and control groups and identify the underlying mechanisms	GT support group had higher GT perspective and life satisfaction than the control group. GT support group can slightly reduce depression in institutionalized elders. The increase of GT perspective found in the support group was greatest in the cosmic transcendence. Although still significant, solitude transcendence showed less change	Satisfaction with life and mental health

### Intervention Characteristics

The interventions under study were performed in groups, except one that involved individual meetings with each participant to complete a life map. According to the articles, all studies reviewed incorporated weekly contact with participants, with sessions ranging from 25 to 120 minutes. Table 1 highlights characteristics of the interventions based on the TIDieR.

Some studies included in the review examined interventions with structured sessions (*n* = 5), while no specific structure was identified for others (*n* = 3). The structured interventions distinguished each session’s content in an organized way for the discussions with participants: that is, they were described as containing beginning, development, and conclusion phases. In that sense, studies that assessed interventions that involved physical activity, artwork, or a life map were considered unstructured for the purpose of this analysis because they contained only one stage of intervention and/or activity.

The interventions with structured sessions in the studies reviewed varied in program duration, session phases, and session content. Four programs lasted 8 weeks (Chen et al., 2019; Wadensten, 2009; Wadensten & Hagglund, 2006; Wang et al., 2011), and one spanned 6 weeks (Wadensten, 2005). Meanwhile, one program involved sessions broken into four phases—sharing aging perspectives, a gerotranscendence exposition, guided discussion, and feedback (Wang et al., 2011)—and another offered sessions with three phases—knowledge and education about gerotranscendence, interaction activity, and sharing experiences (Chen et al., 2019). The articles describing the other six studies did not specify the number of phases in each session, but in general, they were divided into three segments—presentation/warm-up, discussion, and conclusion (Wadensten, 2005, 2009; Wadensten & Hagglund, 2006).

An analysis of the structured sessions’ content uncovered six themes addressed in discussions with participants. The first, cosmic transcendence, was approached by discussing transcendence as a natural perspective and enhancing participants’ understanding of the cosmic domain, and the second, self-transcendence, was approached by asking participants to re-evaluate and explore themselves from a deeper perspective. Social transcendence, the third theme, was put in focus by discussing and re-evaluating by encouraging participants to discuss and re-evaluate their interpersonal relationships with others and through a presentation of the coherence domain. As the fourth theme, ego integrity was addressed by exploring worries, self-management of life events, and ways to overcome depression and late-life challenges, while end-of-life issues, the fifth theme, was addressed by considering the fear of death, end-of-life perspectives, and establishment of self-confidence in the aging process. The sixth theme, life satisfaction, was managed by reviewing successful aging issues and reflecting on ways to improve life satisfaction. The articles indicated that each theme was explored in all studies by inviting participants to share their aging experience in general and/or to focus on a specific matter; educational elements were incorporated when facilitators presented information regarding gerotranscendence.

The unstructured sessions varied. One article refers to 24 Tai Chi postures performed five times a week for 6 months to promote gerotranscendence (Duan et al., 2016); another refers to using artwork to promote a positive aging process for 2 hours each week for more than 6 years (Stephenson, 2013). Another article describes a study that uses a spiritual life map (Molinari et al., 2013).

### Intervention Outcomes

Three studies in the review assessed interventions using experimental and control groups, with the gerotranscendence level as assessed by specific scales serving as the main outcome measure for pre- and post-intervention evaluations (Chen et al., 2019; Duan et al., 2016; Wang et al., 2011). Three studies gathered data through semi-structured interviews to evaluate the effectiveness of interventions and to determine whether participants considered the thematic encounter meaningful (Wadensten, 2005, 2009; Wadensten & Hagglund, 2006), another relied on participants’ artwork (Stephenson, 2013), and a spiritual life map and an interview were used in yet another study (Molinari et al., 2013). Some investigations also evaluated additional constructs to link gerotranscendence to other factors. Along with gerotranscendence, one study evaluated depression levels using the Geriatric Depression Scale—Short Form (GDS-SF) and assessed life satisfaction using the Life Satisfaction Scale (LSS; Wang et al., 2011). The GDS-SF was also used in another study to assess participants’ depression level, along with a Visual Analogue Scale (VAS) used to identify their satisfaction with the intervention and their perceptions of its usefulness (Chen et al., 2019). In addition, the researchers for one study used blood tests to verify participants’ telomerase activity (TA) levels (Duan et al., 2016).

The selected studies were also classified based on a thematic analysis, which produced seven themes: wisdom, life satisfaction, mental health, personal growth, spirituality, physical health, and purpose in life (Table 1). The “wisdom” theme was assigned to studies associated with outcomes involving life experiences, changes in thinking and feeling that occur in old age, and the maturation process (Stephenson, 2013; Wadensten, 2005; Wadensten & Hagglund, 2006). Studies that used a scale (i.e., the LSS) to assess life satisfaction or that reported an increase in participants’ satisfaction, happiness, well-being, or self-esteem were categorized under “life satisfaction” (Stephenson, 2013; Wang et al., 2011). “Mental health” was used for studies in which mental health issues, such as depression, were assessed using a scale (Chen et al., 2019; Wang et al., 2011), and “personal growth” was the designation for studies that reported participants’ reflections on their lives, particularly their past, their reinterpretations of life events, and new perspectives and insights (Molinari et al., 2013; Stephenson, 2013; Wadensten, 2005, 2009; Wadensten & Hagglund, 2006). The “spirituality” theme encompassed studies that assessed or reported participants’ spiritual matters (Molinari et al., 2013), while “physical health” was reserved for studies that assessed any physical dimension pre- and/or post-intervention (Duan et al., 2016). Finally, the “purpose in life” theme was assigned to studies that reported a purpose or direction in life (Duan et al., 2016; Molinari et al., 2013; Stephenson, 2013).

The main results reported for the studies that used control groups involving structured interventions were that gerotranscendence interventions could increase gerotranscendence levels, especially regarding cosmic transcendence; slightly reduce depression levels (Chen et al., 2019; Wang et al., 2011); and increase life satisfaction (Wang et al., 2011). The main results reported for studies featuring structured interventions that did not use control groups included that participants recognized gerotranscendence aspects of their lives, especially for some specific matters, such as cosmic and coherence issues (Wadensten, 2005). Other findings highlighted the importance of giving older adults the opportunity to remember past situations to encourage them to reflect on their lives (Wadensten & Hagglund, 2006) and to provide deeper thoughts about dreams and life situations (Wadensten, 2009) as part of gerotranscendence interventions. Significant findings of studies involving unstructured interventions included that Tai Chi can improve gerotranscendence, which can, in turn, enhance an individual’s purpose in life and telomerase activity (Duan et al., 2016); that creating art can contribute to life satisfaction, self-esteem, and wisdom, which will influence gerotranscendence levels (Stephenson, 2013); that a spiritual life map is an appropriate tool for assessing gerotranscendence; and that significant events occurring from childhood through individuals’ current age can influence their perspectives on life (Molinari et al., 2013).

### Risk of Bias and Critical Appraisal

Of the eight selected studies, only three followed a RCT design; two studies qualified as high-quality appraisals, one as an appraisal of medium quality, and the other five as very low-quality appraisals (See Appendix 2 for full GRADE table). The quality of these studies was considered low mostly due to their observational design, as such studies are considered to be of low quality to begin with based on their design, which makes them prone to bias (Goldet & Howick, 2013). In addition to observational studies presenting a higher risk of bias than RCTs, the observational studies included in this review also presented a serious to critical level of risk of bias (See Appendix 3 for ROBINS table), according to the analysis, because the main domains that increased their risk of bias were the lack of classification of interventions, no measurement of outcomes, and a high risk for confounding (domains 3, 6, and 1, respectively).

Overall, the risk of bias and critical appraisal results indicated that most of the studies presented true effects likely to be substantially different from the estimated effects (Goldet & Howick, 2013); few studies presented methodologically consistent results (Chen et al., 2019; Duan et al., 2016; Wang et al., 2011), meaning that the effects reported in these studies reflect the actual effects of the studied phenomenon. This review did not include many studies, in which a gerotranscendence intervention was performed, and even fewer studies with a RCT design; addressing these weaknesses could make the results more reliable and less biased and could increase the possibility of replicating the intervention.

## Discussion

This scoping review assessed the size and scope of available research on interventions related to gerotranscendence and their effect on participants’ well-being. Articles highlighting eight studies involving interventions related to gerotranscendence were selected for analysis. Studies were organized according to their intervention structure and content, their study type (RCT or non-RCT), and their outcomes. Some articles described studies on interventions with organized structures for specific discussions in each session, while others highlighted studies that focused on an intervention with only one activity. Moreover, RCT studies presented less risk of bias and a higher quality appraisal compared to observational studies. Furthermore, the findings of many of the studies showed an increase in gerotranscendence levels, especially in the cosmic dimension, and that a positive attitude resulted from discussing gerotranscendence matters.

The unstructured interventions varied according to activity: the intervention in one study involved Tai Chi postures (Duan et al., 2016), the intervention in another incorporated participants’ artwork (Stephenson, 2013), and the intervention in a third study involved a life map followed by an interview (Molinari et al., 2013). The use of only one activity to increase gerotranscendence levels was not recommended by Tornstam (2005), as the author suggested exercises to develop gerotranscendence that encompass all dimensions. Nevertheless, previous studies that investigated the relationship between gerotranscendence and other constructs were able to relate to the theory with single activities (Braam et al., 2006, 2016; Gutiérrez, Tomás, et al., 2018a; Lewin, 2001; Lewin & Thomas, 2001; Melia, 2002), which may support the decisions by those conducting the research to perform unstructured interventions of single activities.

The content of structured interventions were more varied and complex, as the authors chose to combine multiple aspects of gerotranscendence for each encounter. The themes selected for discussion (cosmic transcendence, self-transcendence, social transcendence, ego integrity, end of life, and life satisfaction) are present in either the gerotranscendence theory (ego integrity, cosmic and social transcendence; Jewell, 2014; Tornstam, 2011) or associated constructs (self-transcendence, the end of life, and life satisfaction; George & Dixon, 2018; Read et al., 2014; Tornstam, 2005, 2011). This combination may enable participants to become better informed and discuss core aspects of the theory by sharing their perspectives on each dimension (Wadensten, 2005, 2009; Wadensten & Hagglund, 2006) and may promote reflection on gerotranscendence goals (e.g., increasing life satisfaction and decreasing fear of death), which can make the intervention more effective (Chen et al., 2019; Wang et al., 2011).

The thematic analysis indicated that the interventions’ outcomes were associated with positive indicators and with factors previously associated with gerotranscendence. As most were previously cited by Lars Tornstam, 2005, 2011 in his work on important aspects of gerotranscendence, the appearance of these themes aligns with the author’s theory. The “spirituality” and “physical health” themes were not mentioned by Tornstam; however, other studies have associated these themes with the theory of gerotranscendence (Abreu et al., 2022; Bruyneel et al., 2011; Dalby, 2006; Girard, 2014; Gutiérrez et al., 2018a, 2018b). By analyzing these emerging themes, the assumption can be made that most of the results of gerotranscendence interventions agree with the results of available theoretical and exploratory studies.

The quality appraisals of studies that performed RCTs were significantly different from those conducted on the observational studies. The RCTs presented a higher quality due to the rigorous methodology and lower risk of bias. Even with this advantage, the RCTs related only short-term follow-up with participants, meaning no information is available on the long-term effects of the interventions. The main limitation for observational studies was the high risk of bias, as the studies used a small sample with no pre- and post-tests and no control group, suggesting a lower quality intervention. In that sense, the outcomes presented by the RCTs are considered more consistent and replicable (Chen et al., 2019; Duan et al., 2016; Wang et al., 2011), allowing for a deeper analysis and discussion in this review. However, the observational studies importantly contributed knowledge on how gerotranscendence can be approached and provided ideas for future studies (Molinari et al., 2013; Stephenson, 2013; Wadensten, 2005, 2009; Wadensten & Hägglund, 2006).

The RCT studies with structured sessions presented comparable results (Chen et al., 2019; Wang et al., 2011), claiming that gerotranscendence levels were increased in the experimental group but not in the control group. However, the increases in gerotranscendence levels were not homogenous because most changes occurred in cosmic dimension. Cosmic transcendence was also highlighted in previous studies (Braam et al., 2006, 2016; Sadler et al., 2006), as this dimension is more connected to the idea of transcending a life crisis (Read et al., 2014) and is the most expressive dimension among older adults (Heinz et al., 2017; Tornstam, 2005). Additionally, the cosmic dimension also included a protagonism in the GST Scale, present in 5 of the 10 items on the instrument (Tornstam, 2005). This was the dimension older people identified with and referred to most during qualitative studies; hence, it became the main factor of the gerotranscendence scale, assuming an important part in gerotranscendence theory, which may have influenced the results on the emphasis on the cosmic dimension for the RCT studies. Moreover, the studies also reported a decrease in depression among participants (Chen et al., 2019; Wang et al., 2011) and an increase in life satisfaction (Wang et al., 2011). These outcomes align with Tornstam’s 2005, 2011 findings, evidencing the potential for applying the gerotranscendence theory in practice, though more studies are needed to confirm these associations. The third study presenting a RCT design demonstrated that the weekly Tai Chi encounters increased gerotranscendence levels (Duan et al., 2016).

The use of physical activities as something beneficial to the aging process was already explored by Tornstam (2005), and confirmed by other studies (Gutiérrez et al., 2018a, 2018b). Observational studies affirmed that various activities helped to promote gerotranscendence, such as discussing spiritual factors through a life map (Molinari et al., 2013), doing artwork (Stephenson, 2013), discussing dreams (Wadensten, 2009), and sharing elements of the gerotranscendence theory (Wadensten, 2005; Wadensten & Hagglund, 2006), which may align with Tornstam’s suggested exercised for personal development (Tornstam, 2005). Using the life map to increase gerotranscendence levels was justified by the authors due to its spiritual review characteristics (Molinari et al., 2013). The relationship between gerotranscendence and spirituality was studied and established by other studies, which postulated that an increased spirituality can influence gerotranscendence development (Gutiérrez, Tomás, et al., 2018a; Lewin, 2001; Lewin & Thomas, 2001). Furthermore, reviewing one’s life and reflecting upon past events have contributed to enhancing gerotranscendence as shown by another study (Heinz et al., 2017). However, gerotranscendence has been presented as a complex theory with many characteristics, and the use of a single activity may not encompass all aspects (Jewell, 2014; Rajani & Jawaid, 2015a).

In sum, to increase gerotranscendence in older adults, according to the selected studies, interventions must stimulate participants to share about their aging experience and share their thoughts concerning gerotranscendence topics. The combination of presenting a specific topic related to old age for discussion and having a safe and open space in which to share opinions and experiences appears to be important for older adults’ reflection about gerotranscendence matters, resulting in the development of these aspects (Chen et al., 2019; Wadensten, 2005; Wadensten & Hagglund, 2006; Wang et al., 2011). Notably, seven out of eight interventions were performed with participants as a group, which may be due to the importance of social contact for gerotranscendence development (Tornstam, 2005) and/or to the cost effectiveness of group interventions (Bastien et al., 2004). However, group interventions can also present some issues, such as some participants responding better to the intervention than others and variations in the alliance between facilitator and participant, which can cause different levels of adherence (Alldredge et al., 2021; Fals-Stewart et al., 1993). Therefore, researchers who choose group interventions must be aware of each group’s particularities to minimize the differences so participants can more equally realize the benefits.

## Conclusion

By analyzing the studies that reported on gerotranscendence interventions, this review uncovered that increasing older adults’ level of gerotranscendence seems possible, especially relative to cosmic transcendence, which can potentially promote higher life satisfaction and lower levels of depression. This review paves the way for future studies, as a suggestion, to address older adults’ mental health through gerotranscendence with more consistency. Since the studies represent a brief period (2005–2019), future studies can also investigate whether gerotranscendence theory is inherent to the aging process and whether the theory needs updating for future generations. This scoping review’s findings add to knowledge on interventions dedicated to vulnerable populations, such as older adults with mental health concerns and institutionalized older adults (Khodabakhsh, 2022; Tang et al., 2020; Wassink-Vossen et al., 2022), who have become especially vulnerable due to the pandemic (Gerritsen & Oude Voshaar, 2020; Lekamwasam & Lekamwasam, 2020; Penteado et al., 2020; Vahia, 2020). Hence, mapping these interventions is not only important for adding to the gerotranscendence knowledge but also for exposing promising alternatives to promote older adults’ well-being.

## Supplemental Material

Supplemental Material - How to Promote Gerotranscendence in Older Adults? A Scoping Review of Interventions Click here for additional data file. Supplemental Material for How to Promote Gerotranscendence in Older Adults? A Scoping Review of Interventions by Taiane Abreu, Lia Araújo, and Oscar Ribeiro in Journal of Applied Gerontology
